# Supplementary material for: Radiomic-Based Approaches in the Multi-metastatic Setting: A Quantitative Review
Source: medRxiv. 2024 Jul 5:2024.07.04.24309964. Preprint. [Version 1] doi: 10.1101/2024.07.04.24309964 (PMC11245050; doi:10.1101/2024.07.04.24309964)
Supplement: 1 [file NIHPP2024.07.04.24309964V1-supplement-1.pdf]

medRxiv preprint doi: https://doi.org/10.1101/2024.07.04.24309964; this version posted July 5, 2024. The copyright holder for this preprint (which was not certified by peer review) is the author/funder, who has granted medRxiv a license to display the preprint in perpetuity.
It is made available under a CC-BY 4.0 International license .

Supplemental

Literature Search (Specific Database Queries)

Scopus

- TITLE-ABS (radiomics AND (metastas?s OR intertumor) AND (response OR survival)) AND (LIMIT-TO (SUBJAREA , "MEDI")) AND (LIMIT-TO (DOCTYPE , "ar")) AND (LIMIT-TO (LANGUAGE , "English")) AND (LIMIT-TO (EXACTKEYWORD , "Radiomics"));

PubMed

- (radiomics AND inter lesion heterogeneity);
- (radiomics AND metastases AND interlesion);
- (radiomics AND feature aggregation);

Summary of Sample Sizes (Subgroup Analysis)

**Subgroup**	**Dataset**		
	**TCIA - RADCURE**	**TCIA - CRLM**	**SARC021**
All patients	3188 (2961)	197	545 (325)
Patients with 2+ lesions	2333 (3165)	127	407 (288)
Patients with 3+ lesions	2016 (1883)	64	151 (121)

Table S1: *Summary of sample sizes for various subgroup analyses. For the TCIA - RADCURE and SARC021 datasets, numbers in parentheses represent the number of patients with a primary tumor and with a lung metastasis, respectively.*

Numerical Results

	RADCURE (n=3188)		TCIA-CRLM (n=197)		SARC021 (n=545)	
	Train (Median (95% CI)) (n=2550)	Test (n=638)	Train (Median (95% CI)) (n=157)	Test (n=40)	Train (Median (95% CI)) (n=436)	Test (n=109)
**Largest Lesion**	0.599 (0.570-0.634)	0.593	0.664 (0.601-0.739)	0.658	0.622 (0.579-0.659)	0.619
**Largest Lesion + Number of Mets**	0.595 (0.549-0.630)	0.595	0.653 (0.583-0.733)	0.622	0.621 (0.577-0.659)	0.675
**Smallest Lesion**	0.556 (0.530-0.585)	0.545	0.656 (0.575-0.732)	0.627	0.593 (0.544-0.633)	0.587
**Primary Tumor***	0.656 (0.625-0.683)	0.611	-	-	-	-
**Largest Lung Lesion***	-	-	-	-	0.576 (0.523-0.632)	0.627
**Weighted Average (N-largest)**	0.599 (0.570-0.634)	0.593	0.664 (0.601-0.739)	0.658	0.622 (0.579-0.659)	0.619
**Concatenation**	0.599 (0.570-0.634)	0.593	0.664 (0.601-0.739)	0.658	0.622 (0.579-0.659)	0.619
**Cosine Metrics**	N/A					
**Unweighted Average**	0.600 (0.572-0.645)	0.632	0.675 (0.577-0.752)	0.687	0.605 (0.567-0.653)	0.648
**Weighted Average**	0.607 (0.577-0.640)	0.600	0.665 (0.594-0.737)	0.644	0.618 (0.576-0.662)	0.682

medRxiv preprint doi: https://doi.org/10.1101/2024.07.04.24309964; this version posted July 5, 2024. The copyright holder for this preprint (which was not certified by peer review) is the author/funder, who has granted medRxiv a license to display the preprint in perpetuity.
It is made available under a CC-BY 4.0 International license .

Table S2: *Comparison of feature aggregation methods for all patients, irrespective of number of tumors, in three distinct metastatic settings. The Cox proportional hazards model was used to fit the data. The training set was bootstrapped to generate confidence intervals (CI) for model performance; the metric used for model performance is the Concordance Index (C-Index).*

	**RADCURE (n=2333)**		**TCIA-CRLM (n=127)**		**SARC021 (n=407)**	
	**Train (Median (95% CI)) (n=1868)**	**Test (n=465)**	**Train (Median (95% CI)) (n=102)**	**Test (n=25)**	**Train (Median (95% CI)) (n=323)**	**Test (n=84)**
**Largest Lesion**	0.614 (0.584-0.649)	0.604	0.702 (0.623-0.785)	0.681	0.611 (0.571-0.649)	0.633
**Largest Lesion + Number of Mets**	0.597 (0.572-0.630)	0.587	0.669 (0.583-0.747)	0.609	0.617 (0.573-0.649)	0.677
**Smallest Lesion**	0.597 (0.563-0.628)	0.551	0.658 (0.578-0.741)	0.702	0.618 (0.576-0.674)	0.623
**Primary Tumor***	0.570 (0.536-0.605)	0.595	-	-	-	-
**Largest Lung Lesion***	-	-	-	-	0.605 (0.539-0.667)	0.602
**Weighted Average (N-largest)**	0.613 (0.587-0.642)	0.615	0.727 (0.638-0.826)	0.732	0.603 (0.556-0.659)	0.625
**Concatenation**	0.579 (0.543-0.612)	0.576	0.699 (0.613-0.797)	0.689	0.600 (0.550-0.658)	0.614
**Cosine Metrics**	0.500 (0.500-0.500)	0.500	0.500 (0.500-0.531)	0.500	0.500 (0.500-0.500)	0.500
**Unweighted Average**	0.607 (0.578-0.643)	0.614	0.745 (0.656-0.858)	0.689	0.630 (0.583-0.679)	0.671

medRxiv preprint doi: https://doi.org/10.1101/2024.07.04.24309964; this version posted July 5, 2024. The copyright holder for this preprint (which was not certified by peer review) is the author/funder, who has granted medRxiv a license to display the preprint in perpetuity.
It is made available under a CC-BY 4.0 International license .

**Weighted Average**	0.650 (0.619-0.670)	0.623	0.740 (0.653-0.842)	0.821	0.609 (0.556-0.656)	0.646

Table S3: *Comparison of feature aggregation methods for patients with two or more tumors, in three distinct metastatic settings. The Cox proportional hazards model was used to fit the data. The training set was bootstrapped to generate confidence intervals (CI) for model performance; the metric used for model performance is the Concordance Index (C-Index).*

	**RADCURE (n=2016)**		**TCIA-CRLM (n=64)**		**SARC021 (n=151)**	
	**Train (Median (95% CI)) (n=1613)**	**Test (n=403)**	**Train (Median (95% CI)) (n=51)**	**Test (n=13)**	**Train (Median (95% CI)) (n=121)**	**Test (n=30)**
**Largest Lesion**	0.587 (0.558-0.617)	0.589	0.779 (0.659-0.886)	0.821	0.615 (0.532-0.690)	0.627
**Largest Lesion + Number of Mets**	0.587 (0.557-0.617)	0.582	0.797 (0.674-0.912)	0.804	0.615 (0.525-0.690)	0.672
**Smallest Lesion**	0.578 (0.544-0.609)	0.531	0.744 (0.625-0.849)	0.857	0.630 (0.549-0.719)	0.748
**Primary Tumor***	0.594 (0.566-0.626)	0.590	-	-	-	-
**Largest Lung Lesion***	-	-	-	-	0.622 (0.550-0.704)	0.572
**Weighted Average (N-largest)**	0.606 (0.568-0.637)	0.596	0.793 (0.653-0.909)	0.768	0.636 (0.570-0.693)	0.588
**Concatenation**	0.568 (0.530-0.603)	0.617	0.756 (0.553-0.864)	0.821	0.673 (0.586-0.739)	0.598

medRxiv preprint doi: https://doi.org/10.1101/2024.07.04.24309964; this version posted July 5, 2024. The copyright holder for this preprint (which was not certified by peer review) is the author/funder, who has granted medRxiv a license to display the preprint in perpetuity.
It is made available under a CC-BY 4.0 International license .

**Cosine Metrics**	0.520 (0.496-0.555)	0.516	0.595 (0.456-0.756)	0.554	0.532 (0.473-0.605)	0.625
**Unweighted Average**	0.609 (0.574-0.645)	0.627	0.818 (0.670-0.939)	0.839	0.618 (0.544-0.701)	0.674
**Weighted Average**	0.615 (0.581-0.648)	0.586	0.842 (0.714-0.982)	0.982	0.615 (0.545-0.681)	0.674

Table S4: *Comparison of feature aggregation methods for patients with three or more tumors, in three distinct metastatic settings. The Cox proportional hazards model was used to fit the data. The training set was bootstrapped to generate confidence intervals (CI) for model performance; the metric used for model performance is the Concordance Index (C-Index).*

medRxiv preprint doi: https://doi.org/10.1101/2024.07.04.24309964; this version posted July 5, 2024. The copyright holder for this preprint (which was not certified by peer review) is the author/funder, who has granted medRxiv a license to display the preprint in perpetuity.
It is made available under a CC-BY 4.0 International license .
